# Regionalizing Aquatic Ecosystems Based on the River Subbasin Taxonomy Concept and Spatial Clustering Techniques

**DOI:** 10.3390/ijerph8114367

**Published:** 2011-11-22

**Authors:** Yongnian Gao, Junfeng Gao, Jiongfeng Chen, Yan Xu, Jiahu Zhao

**Affiliations:** 1 State Key Laboratory of Lake Science and Environment, Nanjing Institute of Geography and Limnology, Chinese Academy of Sciences, 73 East Beijing Road, Nanjing 210008, China; E-Mails: gaojunf@niglas.ac.cn (J.G.); woyuna@gmail.com (J.C.); zhaojiahu@live.cn (J.Z.); 2 National Marine Environment Monitoring Center, 42 Linghe Street, Dalian 116023, China; E-Mail: xuyandlls@163.com

**Keywords:** aquatic ecoregion, spatial clustering, Taihu Lake watershed

## Abstract

Aquatic ecoregions were increasingly used as spatial units for aquatic ecosystem management at the watershed scale. In this paper, the principle of including land area, comprehensiveness and dominance, conjugation and hierarchy were selected as regionalizing principles. Elevation and drainage density were selected as the regionalizing indicators for the delineation of level I aquatic ecoregions, and percent of construction land area, percent of cultivated land area, soil type and slope for the level II. Under the support of GIS technology, the spatial distribution maps of the two indicators for level I and the four indicators for level II aquatic ecoregion delineation were generated from the raster data based on the 1,107 subwatersheds. River subbasin taxonomy concept, two-step spatial clustering analysis approach and manual-assisted method were used to regionalize aquatic ecosystems in the Taihu Lake watershed. Then the Taihu Lake watershed was divided into two level I aquatic ecoregions, including Ecoregion I1 and Ecoregion I2, and five level II aquatic subecoregions, including Subecoregion II11, Subecoregion II12, Subecoregion II21, Subecoregion II22 and Subecoregion II23. Moreover, the characteristics of the two level I aquatic ecoregions and five level II aquatic subecoregions in the Taihu Lake watershed were summarized, showing that there were significant differences in topography, socio-economic development, water quality and aquatic ecology, *etc*. The results of quantitative comparison of aquatic life also indicated that the dominant species of fish, benthic density, biomass, dominant species, Shannon-Wiener diversity index, Margalef species richness index, Pielou evenness index and ecological dominance showed great spatial variability between the two level I aquatic ecoregions and five level II aquatic subecoregions. It reflected the spatial heterogeneities and the uneven natures of aquatic ecosystems in the Taihu Lake watershed.

## 1. Introduction

During the recent decades, China’s economy has grown rapidly. However, this rapid growth has been accompanied by seriously deteriorated water quality in large parts of the country’s extensive water systems. Each type of water pollution has had its own specific impact directly or indirectly on aquatic ecosystems. The Chinese government has recognized that the current status of water environmental management based on administrative units couldn’t keep from the deteriorating trend in water quality and aquatic ecosystem, and has set up a special research project named the Major

Science and Technology Program for Water Pollution Control and Treatment to study the new water environmental planning and management strategy based on aquatic ecoregions at the watershed scale. Since the “ecological region” concept was first proposed [1], numerous relevant researches [2–13] have been carried out, and it has been increasingly accepted and adopted in the ecological management by various governments in many counties. In recent decades, aquatic ecoregions have gradually become an international research focus and have been introduced into the water resources management as a basic management unit. Aquatic ecoregions in the United States were delineated based on perceived patterns of a combination of causal and integrative factors including land use, land surface form, potential natural vegetation, and soils [14,15]. Aquatic ecoregions in Australia were delineated based on landscape factors that were most likely to influence aquatic ecosystems [16]. Whittier *et al*. thought that regional patterns in terrestrial characteristics could be used as a framework to monitor, assess and report the health of aquatic ecosystems, and they divide the state of Ohio into five ecological regions using spatial patterns in land-surface form, land use, soil and potential natural vegetation [17]. Hessburg *et al*. conducted an ecoregion classification of the interior Columbia River Basin and vicinity (‘the Basin’), and grouped land units (*i.e.*, 7,496 watersheds of the Basin) that were influenced by the same higher order geology and landform feature, and shared similar areal composition of potential vegetation and climate attributes into 53 ecological subregions using the TWINSPAN procedure [5]. Cohen *et al*. described the partitioning of the Loire basin (105,000 km^2^, France) into hydro-ecoregions tested at the mesohabitat scale [18]. Bryce and Clarke thought that ecoregions had been developed at national and state scales for research and resource management, and stream classification was another method to order the variability of aquatic habitats that spanned spatial scales from microhabitat to valley segment, and they developed landscape-level ecoregions for the upper Grande Ronde River basin in northeastern Oregon, 3,000 km^2^ in area [19]. However, in China, very little research has been conducted on aquatic ecoregion delineation [20–22]. Meng *et al*. discussed the delineation method of basin aquatic ecoregions and theirs application prospects in China [20] and then delineated the Liaohe River basin into 3 level I aquatic ecoregions and 14 level II aquatic subecoregions by methods of multi-indicators overlay analysis and expert judgment with support of GIS technology [21]. Zhou and Zheng delineated all the lakes and reservoirs in China into three Level I and six Level II ecoregion divisions [23]. Huang *et al*. and Li *et al*. proposed the general principles, indicators, and methods of aquatic ecoregion delineation at watershed scale (AEDWS) and provided basic information for the establishment and improvement of the theories and approach of AEDWS in China [24,25]. Gao and Gao discussed the hierarchical framework and delineation method of watershed-scale aquatic ecoregion delineation, and delineated the Taihu Lake watershed into two level I aquatic ecoregions based on elevation indicator using GIS overlay analysis method [26]. In this paper, the regionalizing principles, regionalizing indicators and regionalizing method for aquatic ecosystems in the Taihu Lake watershed will be discussed, and on the guide of the theory of regionalizing aquatic ecosystems, driving factor analysis approach will be used to delineate the level I and II aquatic ecoregions in the Taihu Lake watershed based on the river subbasin taxonomy concept and spatial clustering techniques.

## 2. Material and Methods

### 2.1. Study Area

The Taihu Lake watershed is located in the Yangtze River Delta, the south of the Yangtze River, the west of the East China Sea, the north of Qiantang River, and the east of Tianmu and Mao Mountains. It includes parts of Jiangsu, Zhejiang and Anhui Provinces and the Shanghai municipality. The watershed is wide from east to west and narrow from north to south. Its area extends from 119°3′1″E to 121°54′26″E and from 30°7′19″N to 32°14′56″N, and the drainage area of the Taihu Lake watershed occupies about 36.9 thousand km^2^ with a water area of about 6,134 km^2^. This watershed area is a typical temperate continental monsoon climate type and belongs to subtropical and warm-temperate transitional zone, the yearly mean temperature is between 15 to 17 °C. The annual precipitation is approximately 1,181 mm. Elevations vary from about 0 to 1,567 m with an average of around 34.36 m. The more than 200 rivers in the Taihu Lake watershed show a Taihu Lake-centered radial distribution. The water in the Taihu Lake watershed, especially Taihu Lake itself, which is one of the three largest freshwater lakes in China, has been seriously polluted. In 2008, Taihu Lake water was moderately polluted, with a nutritional status of moderate eutrophication and the major pollution indicators were NH_3_-N, BOD_5_, COD, COD_Mn_ and DO. The river water around the lake was also moderately polluted and the major pollution indicators were COD_Mn_ and NH_3_-N. This watershed was selected as study area because of its serious water pollution. The government would carry out water pollution control and treatment to improve water quality using aquatic ecoregions as spatial units for aquatic ecosystem management in watershed scale.

### 2.2. Technical Flow

**Figure 1:**
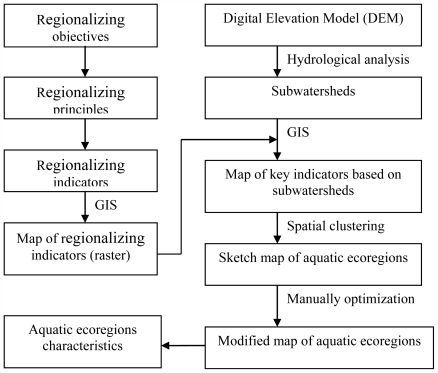
Technical flow to delineate level I and II aquatic ecoregions in the Taihu Lake watershed.

Figure 1 represents the technical flow to delineate level I and II aquatic ecoregions in the Taihu Lake watershed. The regionalizing processing flow mainly includes the following four steps: determination of regionalizing objectives and principles, determination of regionalizing indicators, regionalizing using spatial clustering technique and evaluation of aquatic ecoregions characteristics.

### 2.3. Regionalizing Objectives and Principles

The objective of aquatic ecoregion delineation is to reveal the hierarchical structure and spatial variability of watershed-scale aquatic ecosystems and to provide support for the differentiated management of aquatic ecosystems and the water equality targets management at a watershed scale. The main purpose of level I aquatic ecoregion delineation was to reflect the spatial distribution and pattern of biological species, community and population of aquatic ecosystem in the Taihu Lake watershed, and the purpose of level II aquatic ecoregion delineation was to reflect the spatial variability of diversity and integrity of biological community of aquatic ecosystem in the Taihu Lake watershed.

Regionalizing principles were the basis and criteria of watershed aquatic ecoregion delineation, and determined the rationality and credibility of delineation result. The delineation of level I and II aquatic ecoregions in the Taihu Lake watershed were guided by the following principles:

1. Principle of including land area. Terrestrial ecosystems, climate, geology, soil and other natural conditions as well as human activities in the watershed were the most important factors influencing or determining the composition, structure, pattern, process and function of aquatic ecosystems. In the watershed hydrological processes, a variety of nutrients and pollutants were transported into water and then affected the structure and function of aquatic ecosystems. That is to say, the watershed or subwatershed characteristics could control or influence the aquatic life in rivers, streams and other types of water. Therefore, the land area in subwatershed should be included as part of aquatic ecoregion.
2. Principle of comprehensiveness and dominance. An aquatic ecoregion should not be delineated based only on some of the aquatic ecological components or their driving factors, but rather the comprehensive characteristics of the aquatic ecosystem or their driving factors. In the process of ecoregion delineation, the pattern of various aquatic ecosystem components as well as their similarities and differences of comprehensive features of aquatic ecosystem or their driving factors must be taken fully into account. Based on the comprehensive analysis, the dominant factors influencing the spatial differentiation of aquatic ecosystem should also be considered.
3. Principle of conjugation. The boundaries of the same level aquatic ecoregions did not intersect each other, and the boundaries of adjacent aquatic ecoregions had no space left, and their relationships were seamless and continuous in space. That is to say, each aquatic ecoregion was a complete unit and there was no separation and overlap between each other.
4. Principle of hierarchy. Aquatic ecoregions should have multiple levels such as from level I to level *n*, and be organized in a hierarchical framework and operated at different spatial scales. The watershed aquatic ecosystem should be delineated into multi-level aquatic ecoregions. High-level aquatic ecoregions should contain low-level ecoregions and low-level aquatic ecoregions should be embodied in high-level ecoregions.

In short, the principle of similarity and difference, *i.e.*, keeping the most similarities in structure and function of aquatic ecosystems in the same aquatic ecoregion and the most differences between different aquatic ecoregions, was the fundamental principle to delineate the aquatic ecoregions in the Taihu Lake watershed.

### 2.4. Regionalizing Indicators

The composition, structure, pattern, process and function of aquatic ecosystems were extremely complex. Tens of thousands of different species interact at different levels to produce a balanced system, and the spatial differentiations across aquatic ecosystems were determined by a variety of driving factors, including climate, geology, soil, topography and landforms, as well as human activities. The regionalizing indicators should be able to reflect the potential spatial differentiations across aquatic ecosystems in the Taihu Lake watershed. Thus, the indicators for delineating level I and II aquatic ecoregions in the Taihu Lake watershed were established in view of the effects of driving factors based on the characteristics of the Taihu Lake watershed, as well as the regionalizing objectives and principles, as shown in Table 1.

### 2.5. Regionalizing Using Spatial Clustering Technique

**Figure 2:**
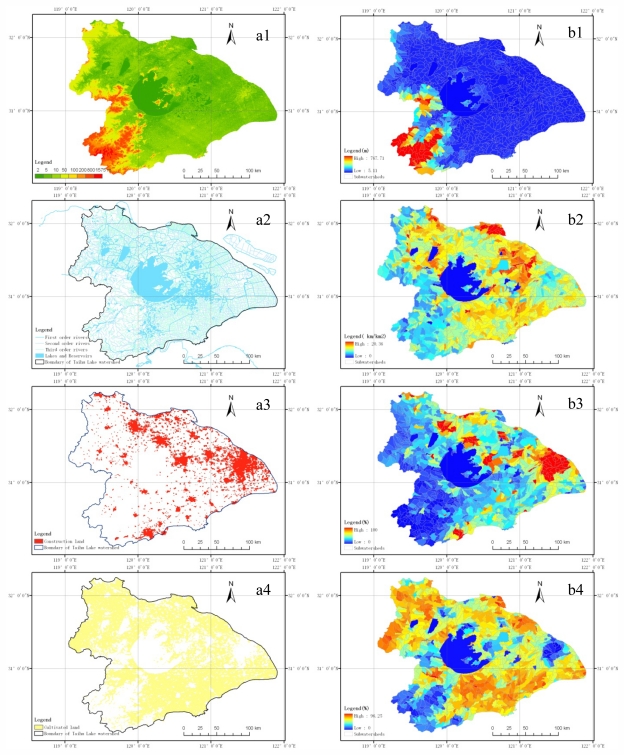

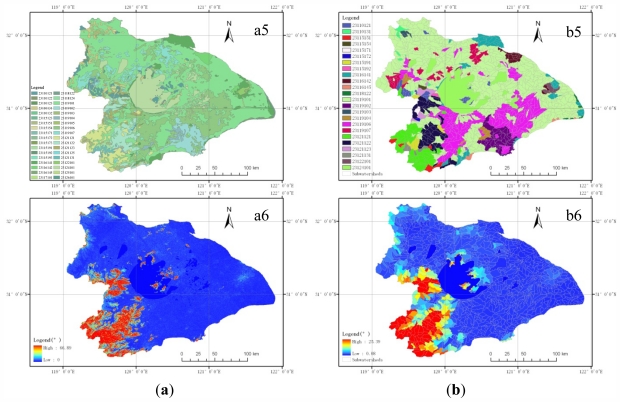
Spatial distribution maps of average elevation (a1 and b1), drainage density (a2 and b2), percent of construction land area (a3 and b3), percent of cultivated land area (a4 and b4), soil type (a5 and b5) and slope (a6 and b6) of 90 × 90 m raster units (**a**) and 1,107 subwatersheds (**b**) in the Taihu Lake watershed.

The data involved mainly included SRTM DEM data with pixel spatial resolution of 90 m, land use map, soil type map, water distribution map, slope data and the Taihu Lake watershed boundary data. The data processing flow mainly included the following three steps:

1. *Delineating Subwatersheds.* Subwatersheds were used as basic clustering units for delineating level I and II aquatic ecoregions in the Taihu Lake watershed. The SRTM DEM data with pixel spatial resolution of 90 m was used to delineate the subwatersheds in the Taihu Lake watershed. A watershed analysis on the terrain model for the Taihu Lake watershed was performed to generate data on flow direction, flow accumulation, stream definition, stream segmentation and watershed delineation using hydrology analysis tool. After the above several processing steps, sketch maps of subwatersheds were obtained. However, the subwatersheds delineated by the hydrology module were inconsistent with the actual stream networks, so further manually-assisted modification was carried out based on the distribution of DEM and the actual river networks. Thus 1,107 small subwatersheds were obtained based on surface drainage patterns.
2. *Mapping the Spatial Distribution of Regionalizing Indicators.* The 1,107 subwatersheds in the Taihu Lake watershed were used as basic calculation units for each indicator. Based on the raster data of regionalizing indicators, the spatial distribution maps of the six key regionalizing indicators, including elevation, drainage density, percent of construction land area, percent of cultivated land area, soil type and slope, were produced using the Zonal Statistics Tool, which calculated statistics on values of raster data within each subwatershed. Figure 2 represents the spatial distribution maps of average elevation, drainage density, percent of construction land area, percent of cultivated land area, soil type and slope of 90 × 90 m raster units and 1,107 subwatersheds in the Taihu Lake watershed.
3. *Spatial Clustering and Manual-Assisted Optimization.* Spatial clustering, which groups similar spatial objects into classes, is an important component of spatial data mining, and it can be used in the identification of areas of similar land usage in an Earth observation database or in merging regions with similar weather patterns, *etc.* [27]. Spatial clustering exceeds the ability of traditional multivariate cluster analysis technique. The two-step cluster method is a scalable cluster analysis algorithm designed to handle very large data sets, and it can handle both continuous and categorical variables. The two-step cluster analysis approach has the advantage of automatically determining the optimal number of clusters according to the clustering criterion with a rapid computation speed and less subjectivity and randomness. The two-step cluster analysis algorithm contains two stages: (1) preclustering and (2) hierarchical clustering. The precluster stage groups the respondents into several small clusters. The cluster stage uses the small clusters as input and groups them into larger clusters.

The spatial distribution maps of the six regionalizing indicators based on subwatersheds, shown in Figure 2(b), were selected and identified as input independent categorical (e.g., soil type) and continuous variables (e.g., elevation and slope), and then the two-step cluster analysis procedure was used to classify and group the six independent variables based on the log-likelihood distance measure and the Bayesian Information Criterion (BIC). However, according to only the results of automatic clustering, the spatial distribution of clusters could not meet the requirements of ecoregion delineation, because parts of the subwatersheds belonging to some clusters were scattered throughout the watershed or in other clusters, and the scattered subwatersheds could not form an independent aquatic ecoregion, so manually-assisted adjustments were required in accordance with the principle of proximity, and the scattered subwatersheds were manually grouped into adjacent clusters with large areas, then the level I aquatic ecoregions and level II aquatic subecoregions were obtained.

### 2.6. Aquatic Life Survey and Evaluation

In this study, a total of seventy-eight water and benthos sampling sites were set and the sampling sites were randomly distributed in the main rivers, lakes and reservoirs. The water and benthos were sampled at the seventy-eight sampling sites in the Taihu Lake watershed on 20 April–10 May and 5–25 July 2010. The benthos samples were collected with 1/40 m^2^ improved Peterson grab devices. After the mud-like materials were removed with sixty mesh nylon screen and placed on white porcelain plates, all the benthic animal specimens were singled out by naked eye inspection, and then preserved in 10% formalin solution. In the laboratory, the specimens were identified to the lowest possible taxas level. After taxas the calculated and weighed, they eventually were converted into density per unit area and wet weight biomass. In this survey, the species and biomass of benthic animals were identified and determined by reference to the criterion of eutrophic lake survey edited by Jin and Tu [28]. A stratified random fish sampling design was used to cope with the uneven distribution of fishes. The multi-mesh gill nets had been designed and used for catching all types of freshwater fish species, and each gill net was composed of 12 different mesh-sizes ranging from 5 mm to 55 mm (knot to knot). Fish sampling was accomplished by reference to the EESTI standard (EVS-EN 14757:2005); more details on the criterion for water quality-sampling of fish with multi-mesh gillnets can be found in [29].

The aquatic ecological survey data were used to validate the rationality of the aquatic ecoregion delineation results. The involved aquatic life validation indicators mainly included fish and benthos in rivers, streams, reservoirs and lake in the Taihu Lake watershed. The fish indicators mainly included the dominant species of fish, while the benthos indicators included mainly density, biomass, dominant species, Shannon-Wiener diversity index *H′*, Margalef species richness index *D*, Pielou evenness index *J* and ecological dominance λ. The Shannon-Wiener diversity index *H′*, Margalef species richness index *D*, Pielou evenness index *J* and ecological dominance λ can be, respectively, expressed as [30,31]:

(1) $$H^{\prime }=-\sum_{i=1}^{s}P_{i}\;\text{ln}\;P_{i}$$

(2) D=(s-1)/ln N

(3) $$J=H^{\prime }/{\text{log}}_{2}\;s$$

(4) $$\lambda =\sum_{i=1}^{s}N_{i}(N_{i}-1)/N(N-1)$$

where, *s* is the total number of species in a sample, *P_i_* is the observed proportion of individuals in sample that belong to species *i* (*i* = 1, 2, …, *s*), *N_i_* is the number of individuals of the species in the sample, and *N* is the total number of individuals of all the species in the sample.

## 3. Results

### 3.1. Aquatic Ecoregions Scheme

**Figure 3:**
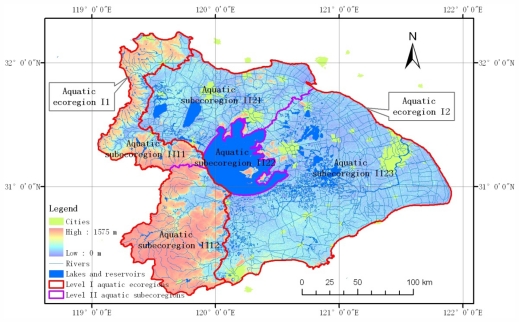
Level I aquatic ecoregions and level II aquatic subecoregions in the Taihu Lake watershed.

According to the above regionalizing method, the Taihu Lake watershed was divided into two level I aquatic ecoregions, Ecoregion I1 and Ecoregion I2, and five level II aquatic subecoregions, including Subecoregion II11, Subecoregion II12, Subecoregion II21, Subecoregion II22 and Subecoregion II23. Figure 3 is the map of level I aquatic ecoregions and level II aquatic subecoregions in the Taihu Lake watershed.

### 3.2. Aquatic Ecoregions Characteristics

1. *General Characteristics.* The two level I aquatic ecoregions and five level II aquatic subecoregions had different aquatic ecosystems, natural resources conditions and socio-economic development characteristics, as shown in Tables 2 and 3.
2. *Aquatic Life Characteristics.* To further validate and assess the scientific, reliability and validity of regionalizing aquatic ecosystems, various indicators of aquatic life, including the dominant species of fish, benthic density, biomass, dominant species, Shannon-Wiener diversity index, Margalef species richness index, Pielou evenness index and ecological dominance, were used to quantitatively compare the spatial differentiations across aquatic ecosystems in different level I aquatic ecoregions and level II aquatic subecoregions.

In the normal season, the dominant species of fish included *Carassius auratus* and *Hemiculter leucisculus* in Ecoregion I1, not only *Carassius auratus* and *Hemiculter leucisculus* but also Pseudorasbora and *Cyprinus carpio* in Ecoregion I2 (Table 4). In wet season, the dominant species of fish in Ecoregion I1 included *Carassius auratus*, *Hemiculter leucisculus* and *Cyprinus carpio*, besides *Sinibrama macro* (Table 4). The benthic density was 5,544 ind/m^2^ and benthic biomass was 204.82 g/m^2^ and dominant species included *Bellamya aeruginosa* and *Chironomus plumosus* in Ecoregion I1. The benthic density and benthic biomass were 3,777 ind/m^2^ and 117.61 g/m^2^ respectively, and dominant species were *Limnodrilus hoffmeister* and *Bellamya aeruginosa* in Ecoregion I2 (Table 5). It can be seen that the fish and benthic animals displayed high variability between two level I aquatic ecoregions.

The benthic density was 12.435 ind/m^2^ and benthic biomass was 475.33 g/m^2^ and dominant species included *Bellamya aeruginosa* and *Limnodrilus hoffmeisteri* in Subecoregion I11, the benthic density was 376 ind/m^2^ and benthic biomass 1.94 g/m^2^ and the dominant species *Limnodrilus hoffmeisteri* and *Chironomus plumosus* in Subecoregion I12, the benthic density 8,339 ind/m^2^ and benthic biomass 56.19 g/m^2^ and dominant species *Limnodrilus hoffmeisteri* and *Bellamya aeruginosa* in Subecoregion I21, the benthic density 45 ind/m^2^ and benthic biomass 0.13 g/m^2^ and dominant species *Limnodrilus hoffmeisteri* and nais sp. in Subecoregion I22, the benthic density 2,117 ind/m^2^ and benthic biomass 167.90 g/m^2^ and dominant species *Limnodrilus hoffmeisteri* and *Bellamya aeruginosa* in Subecoregion I23 (Table 5). It can be found that the benthic density, biomass and dominant species had high variability between five level II aquatic subecoregions.

**Figure 4:**
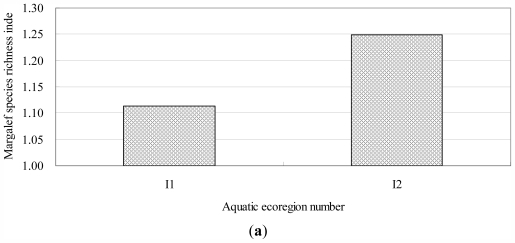

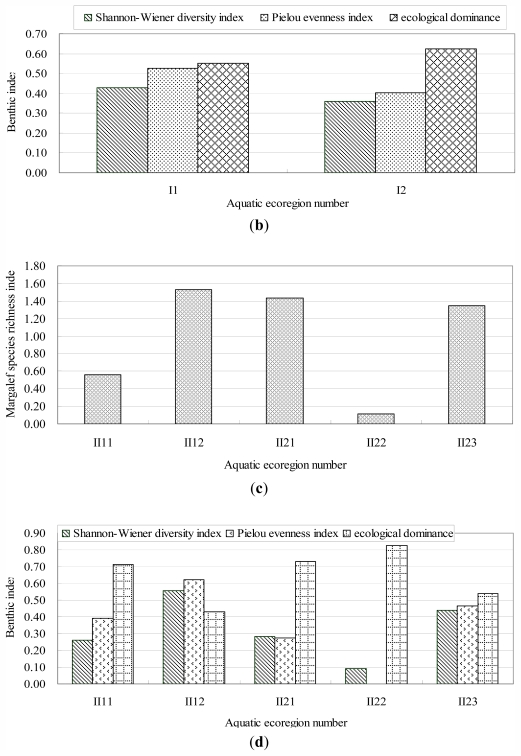
Benthic index in different aquatic ecoregions and subecoregions in Taihu Lake watershed. (**a**) Margalef species richness index in level I aquatic ecoregions; (**b**) Shannon-Wiener diversity index, Pielou evenness index and ecological dominance in level I aquatic ecoregions; (**c**) Margalef species richness index in level II aquatic subecoregions; (**d**) Shannon-Wiener diversity index, Pielou evenness index and ecological dominance in level II aquatic subecoregions.

From the Figure 4, it can be found that the benthic Margalef index in Ecoregion I2 was 1.12 times bigger than that in Ecoregion I1. The Shannon-Wiener diversity index, Pielou evenness index and ecological dominance in Ecoregion I1 were 1.20, 1.31 and 0.88 times bigger than those in Ecoregion I2 respectively. The benthic Margalef indexes were 0.5557, 1.5328, 1.4297, 0.1150 and 1.3488 in subecoregion II11, II12, II21, II22, and II23 respectively. It can be easily found that subecoregion II12 had the biggest Margalef index value and subecoregion II22 had the smallest value. It can be seen that subecoregion II12 had the biggest Shannon-Wiener index, Pielou index and the smallest ecological dominance value, subecoregion II22 had the biggest ecological dominance value and the smallest Shannon-Wiener index according to the comparison of Shannon-Wiener index, Pielou evenness index and ecological dominance between different subecoregions. From the above analysis and Figure 4, it can be easily found that the benthic characteristics showed significant differences between the different subecoregions.

The analysis results of field observed data indicated that the various indicators of aquatic life showed great variability between different ecoregions and subecoregions, shown in Table 4, Table 5 and Figure 4. It illustrated that the two level I aquatic ecoregions and five level II aquatic subecoregions reflected the spatial differentiations and heterogeneity of aquatic ecosystems across the watershed.

## 4. Discussion and Conclusions

Based on the delineation of level I aquatic ecregions and level II aquatic subecregions in Taihu Lake watershed and the comparison tests, the following three conclusions were reached:

1. The level I aquatic ecregions and level II aquatic subecregions in the Taihu Lake watershed were delineated using the 1,107 subwatersheds as the basic clustering units based on the principle of including land area from the view of effects of driving factors. It reflected the land surface physical processes influencing the water quality and aquatic life under the action of the overland flow. The regionalizing method based on spatial clustering technique was feasible in the delineation of level I aquatic ecregions and level II aquatic subecregions in the Taihu Lake watershed.
2. Using the river subbasin taxonomy concept and spatial clustering approach to delineate the level I aquatic ecregions and level II aquatic subecregions in the Taihu Lake watershed was operable and acceptable. In the delineation process, the impacts of people’s subjective experiences or ideas were minimized to the greatest extent. Compared with the traditional regionalizing method based on indicators’ GIS overlay analysis, the proposed regionalizing method using spatial clustering technique based subwatersheds had the advantages of convenience and automation.
3. The Taihu Lake watershed could be delineated into two level I aquatic ecoregions, including Ecoregion I1 and Ecoregion I2, and five level II aquatic subecoregions, including Subecoregion II11, Subecoregion II12, Subecoregion II21, Subecoregion II22 and Subecoregion II23.

The two level I aquatic ecoregions and five level II aquatic subecoregions had obvious spatial differentiations in topography, socio-economic development, water quality and aquatic life, *etc*. The the results of quantitative comparison of aquatic life also indicated that the dominant species of fish, benthic density, biomass, dominant species, Shannon-Wiener diversity index, Margalef species richness index, Pielou evenness index and ecological dominance showed great spatial variability between the two level I aquatic ecoregions and five level II aquatic subecoregions. It reflected the spatial differentiations, heterogeneities and the uneven natures of aquatic ecosystems in the Taihu Lake watershed.

However, only the level I aquatic ecoregions and level II aquatic subecoregions were delineated in this paper, so more work is needed in the future, such as a sensitivity analysis to evaluate the effect of designating 800 or 1,300 subwatersheds, and the sensitivity of the methods presented in the paper and further real tests of the significance of the results, and the statistical analysis of physical characteristics and socio-economic index and stream water quality, as well as the establishment of the basic ideas and goals of aquatic ecological protection in the different delineated ecoregions. Moreover, further research should be focused on the delineation of level III and IV aquatic ecoregions to form a complete hierarchical framework of aquatic ecoregion system in the Taihu Lake watershed.

## Figures and Tables

**Table 1:** Indicators for delineating level I and II aquatic ecoregions in Taihu Lake watershed.

Regionalizing levels	Regionalizing indicators	The role of indicators
Level I	Drainage density	To reflect the potential effects of spatial heterogeneous distribution of surface water resources on aquatic ecosystems.
	Elevation	To reflect the potential effects of regional terrain, which decides the spatial distribution of various factors such as precipitation, temperature, surface runoff, and other macro-scale factors in the Taihu Lake watershed, on the spatial variations of watershed aquatic ecosystems.
Level II	Percent of construction land area	To reflect the potential effects of point source and life diffused pollution load intensity on aquatic ecosystems.
	Percent of cultivated land area	To reflect the potential effects of agricultural non-point source pollution load intensity on aquatic ecosystems.
	Soil type	To reflect the potential effects of spatial distribution of soil types heterogeneity on aquatic ecosystems.
	Slope	To reflect the potential scour strength effects of undulating terrain, influencing the transport of nutrients and pollutants caused by land use activities and soil conditions, on aquatic ecosystems.

**Table 2:** General characteristics of the two level I aquatic ecoregions in Taihu Lake watershed.

Item	Aquatic ecoregion I1	Aquatic ecoregion I2
Total area	The total area is 11.0 thousand km^2^, accounting for 29.81% the total area of the Taihu Lake watershed	The total area is 25.9 thousand km^2^, accounting for 70.19% the total area of the Taihu Lake watershed
Involved administrative regions	Zhenjiang, Changzhou, Nanjing, Wuxi, Xuancheng, Huzhou, Hangzhou	Zhenjiang, Changzhou, Wuxi, Suzhou, Shanghai, Jiaxing, Huzhou, Hangzhou

Topography	Undulating surface, elevation rangs from 0 to 1,575 m with an average of 103.22 m, mean slope is 6.8° with a range difference of 66.89°. The area that elevation equal to and height above 10 m accounts for 73.97% the total area of aquatic ecoregion I1.	Flat surface with scattered hills, elevation rangs from 0 to 351 m with an average of 5.56 m, mean slope is 0.85° with a range difference of 39.79°. The area that elevation is less than 10 m accounts for 95.15% the total area of aquatic ecoregion I2

socioeconomic condition	The relatively smaller population density, slower industrial and agricultural development, and lower economic level, the main land use types are forest land and cultivated land	The bigger population density, the faster industrial and agricultural development and the higher economic level, the main land use types are construction land and irrigated cultivated land

Water quality	The surface water quality is relatively better, mainly belonging to Grade II and Grade III according to the Chinese environmental quality standards for surface water (GB 3838-2002)	The surface water was seriously polluted with a poor water quality, mainly belonging to Grade IV, Grade V and Worse than Grade V according to the Chinese environmental quality standards for surface water (GB 3838-2002).
Aquatic life	The best living conditions for aquatic life, with the nutritional status of mild eutrophication in some large lakes and reservoirs in the region, dominated by *Carassius auratus* and *Hemiculter leucisculus* fish species, tubificida and chironomidae on benthos. There are *Limnodrilus hoffmeisteri*, *Glyptotendipes lobiferus*, *Bellamya aeruginosa*, *Branchiura sowerbyi*, *Limnodrilus grandisetosus*, *etc.*	The worst living conditions for aquatic life, with the nutritional status of moderate eutrophication in some large lakes and reservoirs in the region. There are more than 60 fish species and 60 benthic macroinvertebrates species, dominated by *Corbicula fluminea*, *Limnodrilus hoffmeisteri* and *Stenothyra glabra*, *etc.*

**Table 3:** General characteristics of the five level II aquatic subecoregions in Taihu Lake watershed.

Item	Aquatic subecoregion II11	Aquatic subecoregion II12	Aquatic subecoregion II21	Aquatic subecoregion II22	Aquatic subecoregion II23
Total area	The total area is 4.3 thousand km^2^, accounting for 11.65% the total area of the Taihu Lake watershed	The total area is 6.9 thousand km^2^, accounting for 18.70% the total area of the Taihu Lake watershed	The total area is 8.7 thousand km^2^, accounting for 23.58% the total area of the Taihu Lake watershed	The total area is 2.4 thousand km^2^, accounting for 6.50% the total area of the Taihu Lake watershed	The total area is 14.6 thousand km^2^, accounting for 39.57% the total area of the Taihu Lake watershed
Involved administrative region	Zhenjiang, Changzhou, Nanjing, Wuxi, Xuancheng,	Huzhou, Hangzhou, Xuancheng	Zhenjiang, Changzhou, Wuxi, Suzhou	Changzhou, Wuxi, Suzhou	Wuxi, Suzhou, Shanghai, Jiaxing, Huzhou, Hangzhou

Topography	Undulating surface, elevation ranges from 0 to 586 m with an average of 37.62 m, mean slope is 3.04° with a range difference of 41.30°	Undulating surface, elevation ranges from 0 to 1,575 m with an average of 140.92 m, mean slope is 8.55° with a range difference of 66.89°	Flat surface with scattered hills, elevation ranges from 0 to 353 m with an average of 5.96 m, mean slope is 1.00° with a range difference of 35.27°	Water surface with a small hill, elevation ranges from 0 to 324 m with an average of 1.57 m, mean slope is 0.33° with a range difference of 31.91°	Relative flat surface with scattered hills, elevation ranges from 0 to 331 m with an average of 5.55 m, mean slope is 0.79° with a range difference of 39.79°

socioeconomic condition	The fourth biggest population density (more than 500 persons per sq km), the slowest industrial and agricultural development and the lowest economic level, the main land use types are cultivated land an forest land	The third biggest population density (more than 600 persons per sq km), the third fastest industrial and agricultural development and the third highest economic level, the main land use types are forest land and cultivated land	The second biggest population density (more than 1,000 persons per sq km), the faster industrial and agricultural development and the higher economic level, the main land use type is construction land and irrigated cultivated land	the main land use type is water body	The biggest population density (more than 2,600 persons per sq km), the fastest industrial development and the highest economic level, the main land use ypes are construction land and irrigated cultivated land
Water quality	Relatively good water quality, most belonging to Grade III according to the Chinese environmental quality standards for surface water (GB 3838-2002) with a higher proportion meeting water quality standards. Grade III water environment functional area accounts for 82%, and Grade IV 18%	Relatively good water quality, the Tiaoxi river system and the Sianxi river system have the best surface water quality, and they belong to Grade III except the southern part of the region The best surface water quality, Water quality compliance rate of Dongtiaoxi is 60, and Xitiaoxi 100%, the monitoring sections into Taihu Lake have good water quality, most belonging to Grade III	The surface water was seriously polluted with a poor water quality, mainly belonging to Worse than Grade V and Grade V according to the Chinese environmental quality standards for surface water (GB 3838-2002). The major pollution indicators were NH_3_-N and TN.	The surface water most belongs to the Worse than Grade V with a nutritional status of moderate eutrophication, and high frequency of algal bloom, seriously affecting the safety of drinking water	The surface water was seriously polluted with a poor water quality, mainly belonging to Worse than Grade V and Grade V. Nitrogen and phosphorus pollution load is much higher than the standard of water environment capacity, and the living conditions of aquatic organisms is very poor

Aquatic life	The relatively good living conditions for aquatic life. There are Limnodrilus hoffmeisteri, Glyptotendipes lobiferus, Bellamya aeruginosa, Branchiura sowerbyi, Limnodrilus grandisetosus, *etc.*	The best living conditions for aquatic life, dominated by Carassius auratus and Hemiculter leucisculus among fish species, tubificida and chironomidae in the benthos	The relatively poor living conditions for aquatic life, dominated by Limnodrilus hoffmeisteri and Bellamya aeruginosa in benthos	The relatively poor living conditions for aquatic life, with the nutritional status of moderate eutrophication in the local water body. There are more than 60 fish species and 59 benthic macroinvertebrates species in Taihu Lake, dominated by Corbicula fluminea, Limnodrilus hoffmeisteri and Stenothyra glabra, *etc.*	The relatively poor living conditions for aquatic life, dominated by tubificida and chironomidae and accompanied by planorbidae; trumpet snail, gammaridae and erpobdellidae, *etc.*

**Table 4:** Dominant species of fish in different aquatic ecoregions in the Taihu Lake watershed.

Season	Ecoregion	Species1	Species2	Species3	Species4
Normal	I1	*C. auratus*	*H. leucisculus*		
season	I2	*C. auratus*	*H. leucisculus*	Pseudorasbora	*C. carpio*
Wet	I1	*C. auratus*	*H. leucisculus*	*C. carpio*	
season	I2	*C. auratus*	*H. leucisculus*	*S. macrops*	*C. carpio*

**Table 5:** Characteristics of benthic animals in different aquatic ecoregions and subecoregions in Taihu Lake watershed.

Ecoregions and subecoregions	Benthic density (ind/m^2^)	Benthic biomass (g/m^2^)	Dominant species
I1	5,544	204.82	*B. aeruginosa, C. plumosus*
I2	3,777	117.61	*L. hoffmeisteri, B. aeruginosa*
II11	12,435	475.33	*B. eruginosa, L. hoffmeisteri*
II12	376	1.94	*L. hoffmeisteri, C. plumosus*
II21	8,339	56.19	*L. hoffmeisteri, B. aeruginosa*
II22	45	0.13	*L. hoffmeisteri, Nais sp.*
II23	2,117	167.90	*L. hoffmeisteri, B. aeruginosa*
